# Management of glaucoma with Boston type 1 keratoprosthesis

**DOI:** 10.5935/0004-2749.2022-0216

**Published:** 2025-08-22

**Authors:** Nishaant Bhambra, Mona Harissi-Dagher

**Affiliations:** 1 Faculty of Medicine, McGill University, Montréal, Québec, Canada; 2 Department of Ophthalmology, Université de Montréal, Centre Hospitalier de l’Université de Montréal, Montreal, QC, Canada

Dear Editor,

We read with interest the study “Management of glaucoma with Boston type 1 keratoprosthesis”^(1)^. In this study, the authors retrospectively reviewed and included patients who received Boston keratoprosthesis type 1 (KPro) implantation and had pre-existing or developed de novo glaucoma after surgery. Patients who did not present with or develop glaucoma were excluded in the review.

The authors note that 9 of the 17 patients had glaucoma before or after KPro surgery, with 3 of these patients developing glaucoma after KPro surgery. However, the authors note that these patients had their intraocular pressure (IOP) effectively managed pharmacologically and did not require the implantation of a glaucomatous drainage device (GDD). Of the 17 patients, three received GDD implantation 6 months before or simultaneously with KPro surgery, of whom 100% developed retinal detachment. One of these patients also developed bacterial endophthalmitis. However, none of the three eyes that received GDD implantation years before KPro surgery developed complications other than glaucomatous progression.

The authors conclude that the management of patients with pre-existing glaucoma is more difficult than those with de novo glaucoma after KPro, as 50% of the patients with pre-existing glaucoma or pre-KPro GDD required further glaucoma surgeries. We feel that the small sample size of patients limits the ability to draw these conclusions. While the recent placement of a GDD may have influenced the likelihood of KPro patients then developing retinal detachment, drawing firm conclusions from three patients is not possible. Notably, retinal detachment does occur more commonly in KPro patients, as shown by Jardeleza et al.^(2)^. However, a larger study of KPro implantation in 137 eyes with and without prior GDD placement by Lenis et al. did not show any significant difference in safety outcomes, including rates of retinal detachment^(3)^. Furthermore, as the total number of patients is low (3 patients), no reasonable conclusions can or should be drawn on the sole pharmacological treatment of post-KPro glaucoma.

It is for this reason that we feel that the study here presents too small of a sample size for any reasonable conclusions to be drawn. As no significance testing or statistics were able to be run for such a small sample size, a study as this should be carefully described as a case series, rather than a retrospective study.

We appreciate the authors’ contribution to the field and for the sharing of their findings with the KPro community. We would welcome an expanded study with a larger patient cohort to draw conclusions between patients with de novo and post-KPro glaucoma.
