# Supplementary material for: Management of glaucoma with Boston type 1 keratoprosthesis
Source: Arq Bras Oftalmol. 2025 Aug 22;85(5):540–2. doi: 10.5935/0004-2749.2022-0216 (PMC12372517; doi:10.5935/0004-2749.2022-0216)
Supplement: Related texts

# Reply: Response: management of glaucoma with Boston type 1 keratoprosthesis

**Authors:** Canan Asli Utine

**Affiliations:** 1 Department of Ophthalmology, Dokuz Eylul University, Izmir, Turkey; 2 Izmir Biomedicine and Genome Center, Izmir, Turkey

Dear Editor,

We would like to thank Bhambra and Harissi-Dagher for their interest in our study^(1)^. It has been stated in the manuscript that the study reflects the retrospective results of our own cohort. This study has several limitations, including a small number of cases that render it impossible to perform statistical analysis and a relatively short follow-up. Conclusion was drawn based on our experience, and a scientific hypothesis about the observed differences was formed, which made sense in the peer-review by glaucoma specialists. We would like to emphasize that our conclusion about the safety concern of recent glaucoma drainage device implantation with

KPro surgery includes only aphakic eyes with limited core vitrectomy.

We would be honored to participate in any prospective study on patients with KPro and glaucoma, to find out whether glaucoma drainage devices implanted at least 6 months before aphakic KPro surgery or simultaneous implantation has any clinical differences.

## References

[1] Utine CA, Arıkan G, Yardım E, Günenç Ü. (2022). Management of glaucoma with Boston type 1
keratoprosthesis. Arq Bras Oftalmol [Internet].
